# Loss of Long Distance Co-Expression in Lung Cancer

**DOI:** 10.3389/fgene.2021.625741

**Published:** 2021-03-10

**Authors:** Sergio Daniel Andonegui-Elguera, José María Zamora-Fuentes, Jesús Espinal-Enríquez, Enrique Hernández-Lemus

**Affiliations:** ^1^Computational Genomics Division, National Institute of Genomic Medicine, Mexico City, Mexico; ^2^Centro de Ciencias de la Complejidad, Universidad Nacional Autónoma de México, Mexico City, Mexico

**Keywords:** lung adenocarcinoma, squamous lung cancer, gene co-expression networks, differentiation processes in cancer, loss of distant co-expression

## Abstract

Lung cancer is one of the deadliest, most aggressive cancers. Abrupt changes in gene expression represent an important challenge to understand and fight the disease. Gene co-expression networks (GCNs) have been widely used to study the genomic regulatory landscape of human cancer. Here, based on 1,143 RNA-Seq experiments from the TCGA collaboration, we constructed GCN for the most common types of lung tumors: adenocarcinoma (TAD) and squamous cells (TSCs) as well as their respective control networks (NAD and NSC). We compared the number of intra-chromosome (*cis-*) and inter-chromosome (*trans-*) co-expression interactions in normal and cancer GCNs. We compared the number of shared interactions between TAD and TSC, as well as in NAD and NSC, to observe which phenotypes were more alike. By means of an over-representation analysis, we associated network topology features with biological functions. We found that TAD and TSC present mostly *cis-* small disconnected components, whereas in control GCNs, both types have a giant *trans-* component. In both cancer networks, we observed *cis-* components in which genes not only belong to the same chromosome but to the same cytoband or to neighboring cytobands. This supports the hypothesis that in lung cancer, gene co-expression is constrained to small neighboring regions. Despite this loss of distant co-expression observed in TAD and TSC, there are some remaining *trans-* clusters. These clusters seem to play relevant roles in the carcinogenic processes. For instance, some clusters in TAD and TSC are associated with the immune system, response to virus, or control of gene expression. Additionally, other non-enriched *trans-* clusters are composed of one gene and several associated pseudo-genes, as in the case of the FTH1 gene. The appearance of those common *trans-* clusters reflects that the gene co-expression program in lung cancer conserves some aspects for cell maintenance. Unexpectedly, 0.48% of the edges are shared between control networks; conversely, 35% is shared between lung cancer GCNs, a 73-fold larger intersection. This suggests that in lung cancer a process of de-differentiation may be occurring. To further investigate the implications of the loss of distant co-expression, it will become necessary to broaden the investigation with other omic-based approaches. However, the present approach provides a basis for future work toward an integrative perspective of abnormal transcriptional regulatory programs in lung cancer.

## Introduction

Lung cancer is one of the most deadly types of cancer nowadays. The survival range for lung cancer barely reaches 5.8%, quite below that of other malignant tumors (Torre et al., 2015). The World Health Organization places malignant tumors of the trachea, bronchi, and lung as the sixth leading cause of death globally (Marciniuk et al., 2017). Lung cancer occupies the first place in incidence and worldwide mortality among malignant tumors. Each year there are about 1.8 million new cases and 1.59 million deaths worldwide.

Currently, based on the type of tissue, lung cancer can be classified into two main categories: non-small cell lung cancer (NSCLC) and small cell lung cancer (SCLC). They represent around 80 and 20% of cases, respectively. NSCLC tumors are subclassified into squamous cells (TSC), adenocarcinoma (TAD), and large cell (LC) carcinoma. TSC occurs more frequently in the central area of the lungs, while TAD is found in peripheral areas, arising from bronchial glands and bronchial epithelium (Travis et al., 2015).

Treatment largely depends on histological diagnosis and tumor status. Detection is performed via chest X-ray and low-dose spiral tomography. Currently, only one-third of patients—diagnosed at an early stage—may be candidates for a surgical resection. However, recurrence after surgery reaches 30–60% even with adjuvant chemotherapy. For advanced states, the first line of treatment is chemotherapy with an average response between 30–40%.

### Molecular Biology of Lung Cancer

Several mechanisms of genomic alterations have been found in lung cancer. For instance, DNA-repair pathways are triggered by exposure to tobacco-derived carcinogenic chemicals. Several single nucleotide polymorphisms (SNPs) have been identified in these pathways. The helicase ERCC2/XPD involved in DNA repair, the PHACTR2 protein that regulates the cytoskeleton, the DUSP1 protein that negatively regulates the MAP-kinase pathway are examples where SNPs have been identified (≈25% of cases) in lung adenocarcinoma (Spinola et al., 2007).

In terms of epigenetic marks, alterations have been reported via sputum analysis. In smokers, 14 genes with altered methylation patterns were identified (p16INK4a, DAPK, RASSF1A, PAX5, MGMT, GATA5, among others). These genes were associated with an increase of 50% in the risk of developing lung cancer. On the other hand, the p16 region has been found hypermethylated in 25–74% of lung cancer patients in different studies (Suzuki et al., 2014).

Alternative splicing events have been reported generating gene fusion in lung adenocarcinoma. Tyrosine kinase domain fusions have been identified by sequencing, including dimerization domains, such as EML4-ALK, KIF5B-RET, and CD74-ROS1, among other combinations. Additionally, some of these alterations have been observed to be involved in drug resistance. Patients with the EML4-ALK fusion treated with an ALK tyrosine kinase inhibitors have shown better results than traditional chemotherapy (Campbell et al., 2016). In nonsmoking women from Korea with adenocarcinoma mutations, gene fusions, among other alterations, were identified in c-Ret kinase as well as genes involved in mitotic progression and G2/M transition pathways (Campbell et al., 2016).

Two molecular pathways have been identified as relevant for lung cancer in recent years: the epidermal growth factor receptor (EGFR) and the anaplastic lymphoma kinase (ALK), respectively. These pathways can be affected by mutations in the kinase domain, amplification of the copy number or translocations, thus inducing new transcriptional control. Clinical trials have shown that patients whose malignant tumor is strongly related to EGFR or ALK can be treated with drugs targeting the kinase activities of these proteins, obtaining a 60% favorable response range (Suzuki et al., 2014).

Copy number alterations have also been identified in lung cancer. Chromosomal amplification of region 14q13.3 has been frequently found in tumor adenocarcinoma (TAD). One of the altered genes in copy number is NKX2-1, a transcription factor related to the differentiation and epithelial morphogenesis of the lung.

Several mutations have been reported in crucial genes associated with carcinogenic processes of the lung. KRAS, HER2, BRAF, EGFRvIII, and PIK3CA, among others, are frequently mutated in patients with NSCLC. Mutated KRAS is present in 15–25% of adenocarcinoma cases. There is no directed treatment targeting KRAS, but the subsequent effector route, RAS/ RAF/MEK, possesses inhibitors which may be effective in patients with diagnosed NSCLC and mutant KRAS (Shames and Wistuba, 2014). These are just some examples of the multiplicity of mutations and functional events related to abnormal regulation in lung cancer and its consequences. The purpose of this work is to further contribute to the understanding of these complex phenomena.

### Large-Scale Studies on Abnormal Gene Regulation in Lung Cancer

Several efforts involving next-generation sequencing techniques have been developed by international groups. The objective is to provide a better understanding of the molecular changes that cells and tissues suffer during cancer progression. Endeavors such as The Cancer Genome Atlas (TCGA) or the International Cancer Genome Consortium (ICGC) (Consortium et al., 2010) represent world-wide referents that have broadened our knowledge of cancer.

Collaborations like the ones mentioned above have helped to establish the relevance of cancer genomics and provided large amounts of data that have contributed to improve not only our basic knowledge of cancer biology but also oncological treatment and clinical practice. Data generated by such consortia are public and available to develop new knowledge based on such state-of-the-art experiments for thousands of samples.

A useful and powerful type of data to implement -omic analysis is the one generated by RNA-Seq technology. In the case of lung cancer, TCGA RNA-Seq-based gene expression databases include more than 1,100 samples for patients with adenocarcinoma (533), squamous cell carcinoma (502), as well as their adjacent-to-tumor healthy counterpart samples (101). This kind of information allows researchers to explore in-depth the molecular mechanisms behind each cancerous genotype and at the same time to explore the functional implications of the concomitant phenotypes.

Gene expression data are one of the most used types of genomic information. However, gene expression analysis alone is not always sufficient to fully characterize and differentiate one type of cancer from another, even in the same tissue. Recently (Zamora-Fuentes et al., 2020, published in this research topic), we showed that, for clear cell renal carcinoma, gene expression signatures do not change during cancer progression. However, what remarkably differs between stages is the co-expression signature.

Gene co-expression networks are a helpful tool to analyze not only network parameters to distinguish global features, such as node degree or betweenness centrality between cases, but also functional implications based on the network structure for each phenotype (Amar et al., 2013; Alcalá-Corona et al., 2016, 2017, 2018; Drago-García et al., 2017; van Dam et al., 2018; Fionda, 2019; Tieri et al., 2019).

Despite several efforts to dissect the molecular mechanisms behind lung cancer origins and development, unsolved issues regarding the effect of gene co-expression and the relationship between co-expression patterns and phenotypic manifestations are still missing.

In this work, based on 1,143 gene-expression profiles of NSCLC patients, we constructed, inferred, and analyzed gene co-expression networks of lung cancer, as well as their healthy counterparts. To construct the networks, we separated cancer samples in adenocarcinoma tumors (TAD) and squamous carcinoma tumors (TSC).

We investigated how similar are both types of lung cancer at the expression and co-expression levels. We compared the resulting probabilistic co-expression networks in terms of shared interactions between lung cancer networks (TAD and TSC) and between the healthy ones (NAD and NSC). Finally, based on the gene co-expression signatures for both cancer networks, we performed over-representation analysis to observe those biological processes in which key genes participate.

## Materials and Methods

### Data Acquisition

RNA-Seq files were obtained from the Genomic Data Commons database https://portal.gdc.cancer.gov/ for the two most common subtypes of lung cancer (TAD and TSC) as well as for adjacent-to-tumor normal lung tissue.

Files were downloaded using the following filters: Primary site = lung, sample type = primary tumor or solid normal tissue, experimental strategy = RNA-Seq, and workflow type = HTSeq-Counts. Data files consisted of 502 TAD samples, 49 adjacent-to-TAD normal samples (NAD); 533 TSC samples and 59 adjacent-to-TSC normal samples (NSC).

Data were annotated and harmonized for subsequent analysis using the latest genomic reference (GRCh38). Genomic information for gene stable ID, chromosome/scaffold name, gene start (bp), gene end (bp), gene% GC content, and gene type was mapped using BioMart database (version GRCh38.p12). This data pre-processing pipeline has been previously implemented to analyze RNA-Seq data from breast cancer (Drago-García et al., 2017; Espinal-Enriquez et al., 2017; Dorantes-Gilardi et al., 2020; García-Cortés et al., 2020; Serrano-Carbajal et al., 2020) and clear cell renal carcinoma (Zamora-Fuentes et al., 2020).

### Data Pre-Processing

Quality control was performed using “Biotype detection” and “Sequencing depth” functions from NOISeq package (Tarazona et al., 2011). The most frequent sources of biases in RNAseq sequencing are associated with GC content, transcript length, and RNA composition (Tarazona et al., 2015). These biases were removed using full quantile normalization for GC content and length and TMM (Trimmed Mean of M) for RNA composition, all functions from NOISeq package. In addition, structural noise like batch effects were removed using ARSyN (Nueda et al., 2012) package. Finally, genes with *countspermillion* < 10 were removed. Data pre-processing was carried out using R version 3.6.0.

For data normalization, we used the DESeq2 R package (Love et al., 2014). After normalization of the four matrices, we preserved only those transcripts that were conserved in all four matrices. The number of resulting transcripts was 20,101. This number included protein coding genes, long noncoding RNA, microRNAs, pseudogenes, and other types of RNA species. The whole pre-processing and normalization code can be accessed and/or downloaded from https://github.com/CSB-IG/regulaciontrans-pipeline.

### Differential Expression

Limma (Ritchie et al., 2015) is a Bioconductor component package based on a linear model to compare gene expression between two different gene sets. It can be used to analyze both types of data: microarrays or RNA-Seq. With this tool, we obtained the information about average expression, as well as the differential expression in terms of Log_2_ fold change for TAD vs. NAD, and TSC vs. NSC samples. An absolute difference of fold change ≥ 1.5 and a Benjamini & Hochberg corrected *p-value* < 0.01 were set as thresholds.

### Gene Co-Expression Network (GCN) Inference

For inferring our four GCNs (NAD, TAD, NSC, and TSC), we used mutual information (MI) as the measure to determine gene co-expression. ARACNe (Margolin et al., 2006) is a standard method to calculate the MI between two data series. This algorithm was applied to the four gene expression profiles to establish correlations between pairs of genes. We used the serial C++ version without Adaptive Partitioning Inference.

To improve the performance of this method, we developed a multicore version based on the aforementioned algorithm. This interface accelerates MI calculation depending on the number of available cores. For this work, we inferred a GCN of ≈200, 000, 000 (20, 000^2^/2, corresponding to the total of genes in the matrix) of 100 sample expression matrix in 30 min using an 80-core server. This interface is available on github (https://github.com/josemaz/aracne-multicore). We decided to analyze and conserve the top-10,000 interactions for the four GCNs in order to have the same size of the four graphs, as well as being able to compare them. Additionally, this network size has been previously observed to be significant to analyze them in terms of structural and functional characteristics (Alcalá-Corona et al., 2016, 2017; Velazquez-Caldelas et al., 2019; Zamora-Fuentes et al., 2020).

### *cis-/trans-* Proportion Calculations

Previously, we observed in breast cancer GCNs (Espinal-Enriquez et al., 2017; de Anda-Jáuregui et al., 2019a,b,c; García-Cortés et al., 2020) that gene co-expression interactions occur in a preferential manner between genes from the same chromosome, and inter-chromosome interactions appear more frequently in noncancer breast tissue networks. We decided to observe whether or not that effect is also found in lung cancer networks. For that purpose, we separated gene co-expression interactions between intra-chromosome (*cis-*) and inter-chromosome (*trans-*).

For these analyses, we also used the top-10,000 interactions. However, in order to corroborate that any result generated by the analysis with 10,000 edges network was not related to the network size, we also performed calculations for a range of three orders of magnitude in terms of edges, i.e., we analyzed the *cis-/trans-* proportion in GCNs from 1,000 to 100,000 interactions. Finally, network visualizations and topological analyses were performed using Cytoscape v3.8.1.

We mentioned that the number of cancer samples is much larger than healthy samples. To assure that the obtained results for cancer GCNs were not due to the sample size, we developed a method to select 100 random cancer samples from the cancer expression matrix (table with samples and gene expression). For this work, we generated 10 randomized expression matrices with 100 samples for adenocarcinoma samples and other 10 matrices for squamous cancer data. The networks obtained using this method were pruned to 10,000 interactions, and compared with their healthy counterpart in terms of *cis-/trans-* proportion.

### Functional Enrichment

**Figure 1:**
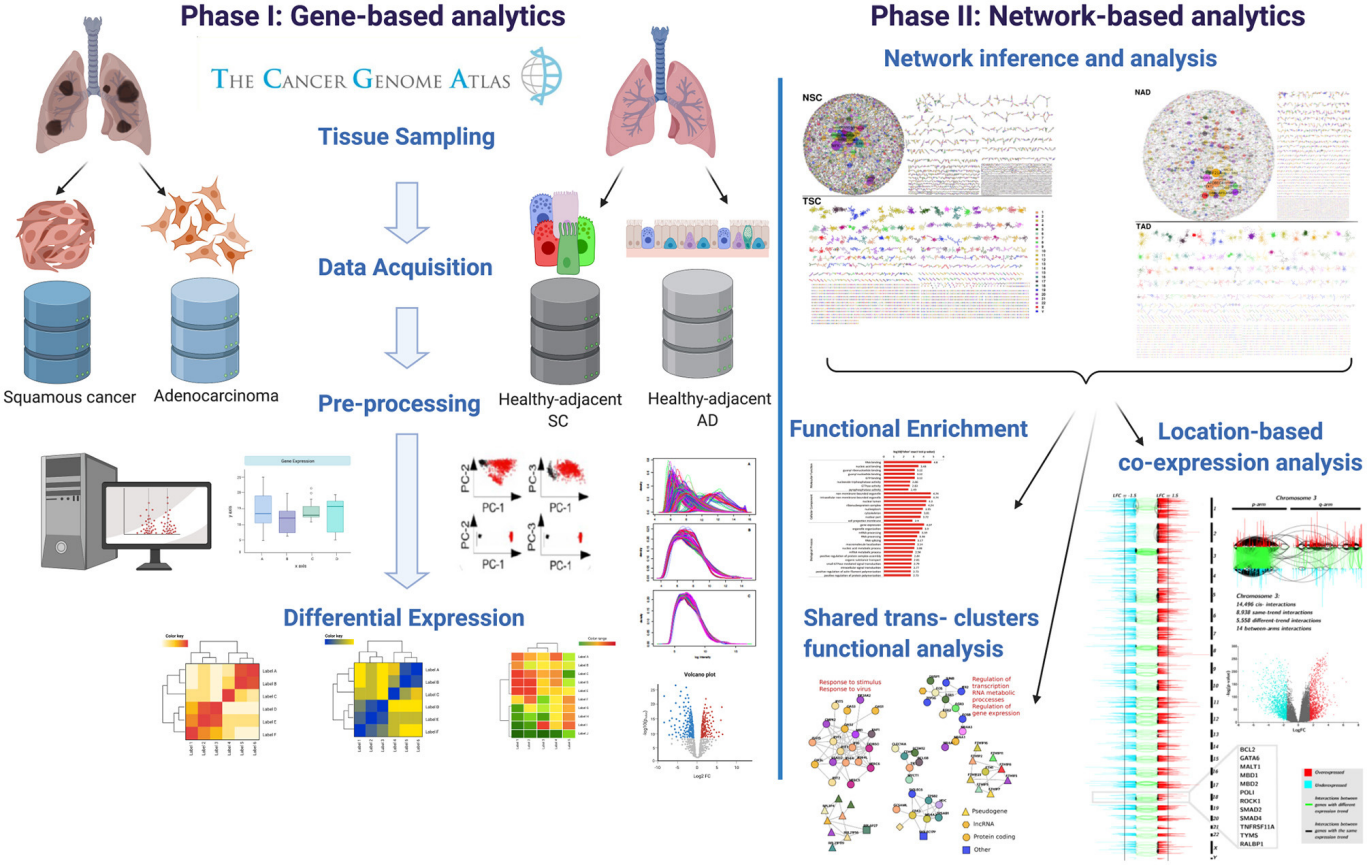
Graphical pipeline followed in this work. In the figure, we show the main steps in which this methodology is divided.

Genes that presented a relevant network topology were in turn mapped into Gene Ontology categories to observe those processes that are allegedly enriched. For that purpose, we used g:Profiler web interface tool (Raudvere et al., 2019). We used the *g:SCS* option for multiple testing correction. The significance threshold was set to 10^−5^. In Figure 1, the workflow presented in this paper is depicted.

## Results and Discussion

### Gene Co-Expression Is Chromosome Dependent in Lung Cancer

**Figure 2:**
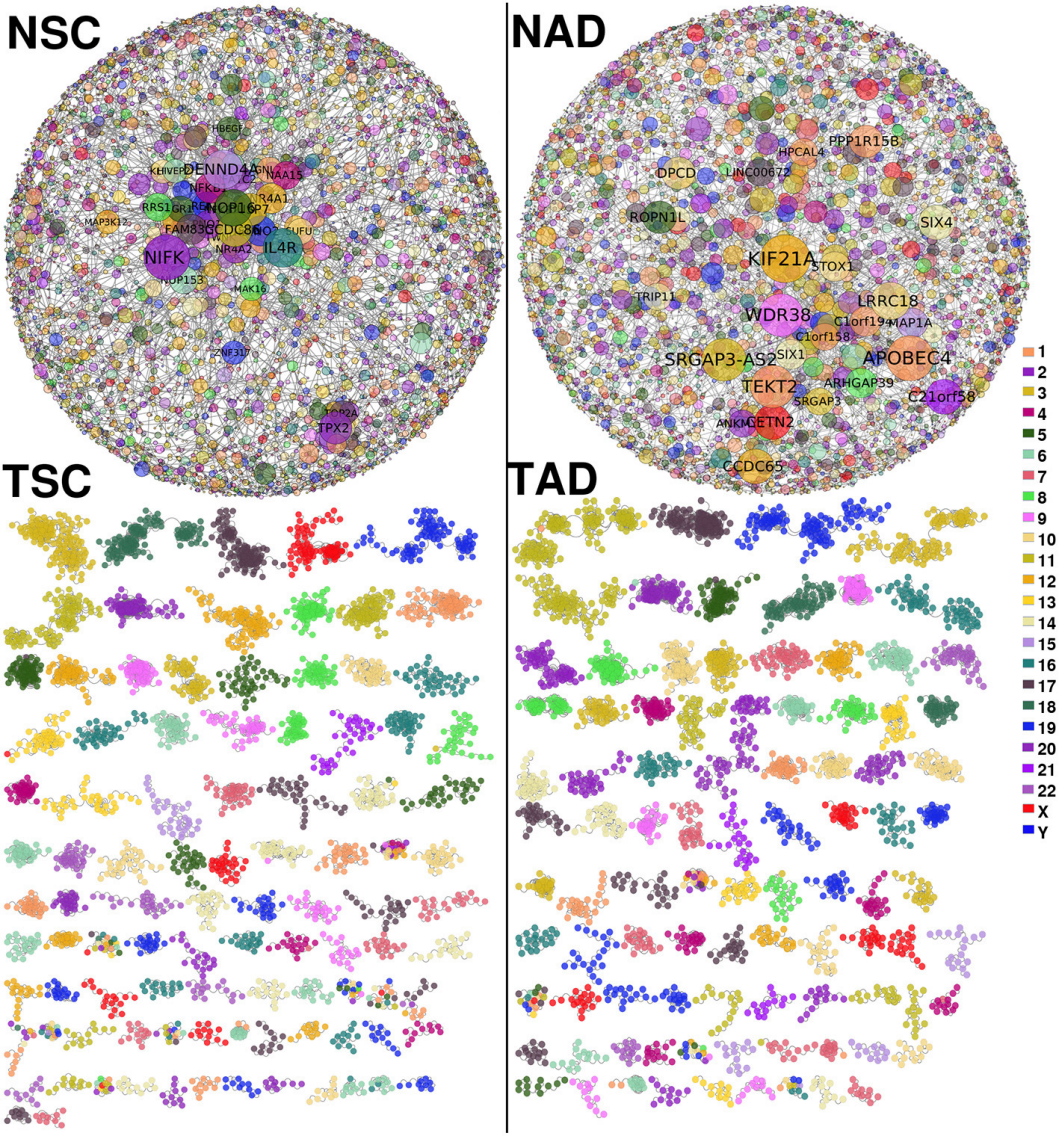
Non-small cell lung cancer (NSCLC) and normal tissue-derived gene co-expression networks. **(Top-left)** Largest component of normal tissue-derived network for NSC. **(Top-right)** Correspond to the giant component of NAD gene co-expression network (GCN). **(Bottom-left)** Squamous carcinoma-derived gene co-expression network. **(Bottom-right)** Tumor adenocarcinoma network. In both tumor GCNs, components with more than 10 genes are depicted. Genes are colored according to the chromosome location. In healthy GCNs, gene size is proportional to the gene connectivity.

Figure 2 shows the lung carcinoma (TSC and TAD) GCNs, compared with their healthy counterpart (NSC and NAD). The difference between both networks in terms of the component sizes is remarkable. The giant component of the healthy GCNs covers more than the half of the total size of the networks. Meanwhile, for the tumor-derived GCNs, there is no giant component; the larger one contains 123 genes and 336 edges (for TSC).

Aside from topological differences in network structure, in the tumor GCNs, components are formed mostly by genes from the same chromosome, which indicates that the majority of interactions are intra-chromosome or *cis-* interactions. Conversely, in the healthy networks genes co-express with other genes with no particular bias or trends in terms of the chromosomal location. The difference in *cis-* and *trans-* interactions between tumor and normal GCNs is observed in all chromosomes (*p*−_*val*_ < 10^−8^ in both cases). In Supplementary Material 1A, we show all *cis-* interactions per chromosome for the four GCNs.

**Figure 3:**
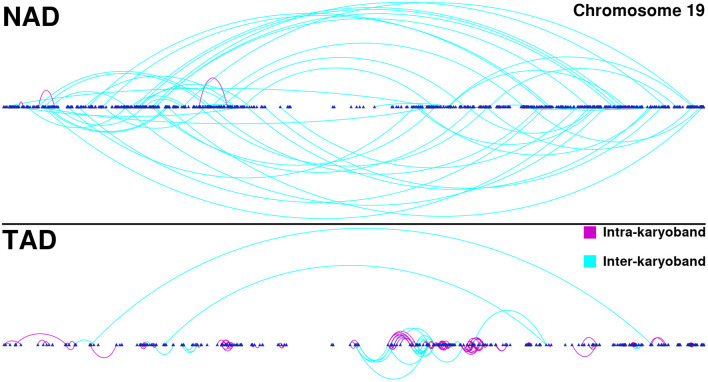
Chromosome 19 gene co-expression networks (GCNs) for NAD and TAD. In this figure, *cis-* interactions for NAD (top) and TAD (bottom) are depicted. Genes are placed according to its starting base pair. Turquoise links join genes from different cytobands, meanwhile purple interactions take account for intra-cytoband relationships.

Furthermore, in the TAD and TSC GCNs, genes are correlated with other genes appearing in the same chromosome, but co-expressed genes are also physically close (in terms of chromosomal location) among them. This phenomenon can be observed in Figure 3. There, we depicted all interactions appearing in chromosome 19 for NAD and TAD GCNs. Genes are placed according to its gene start position. Turquoise interactions represent long-range *cis-* interactions, meanwhile purple edges show close co-expression relationships (both genes belong to the same cytoband).

### Potential De-Differentiation of the Gene Co-Expression Program in Lung Cancer

Since both healthy and both cancer networks at first sight seem to be topologically similar, we decided to compare them in terms of shared genes an interactions: NAD vs. NSC and TAD vs. TSC. This was made out with the aims of observing the percentage of similarity between phenotypes.

**Figure 4:**
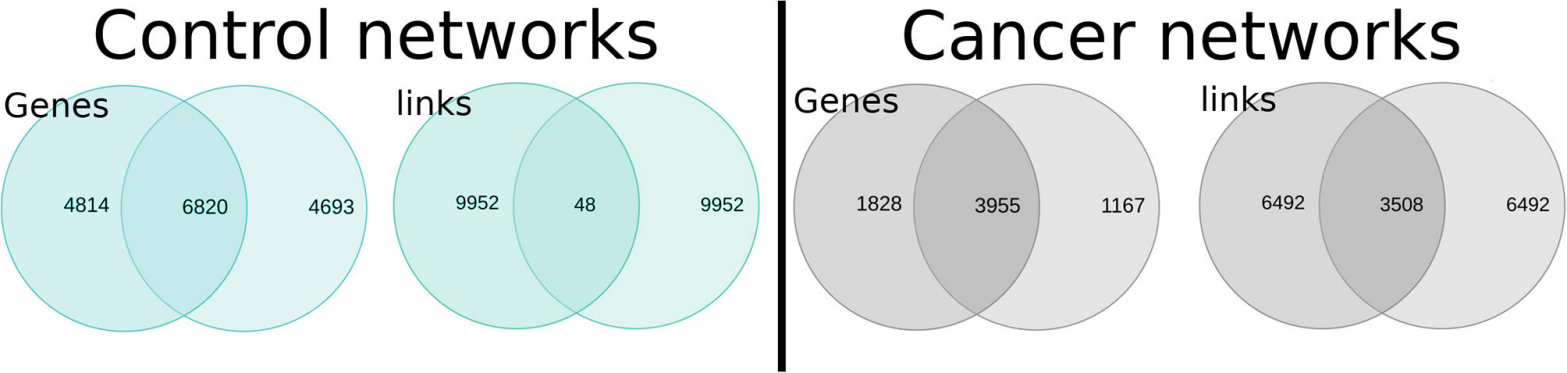
Intersections between normal **(Left)** and lung cancer **(Right)** gene co-expression networks (GCNs). The upper section of the figure shows (in the form of Venn diagrams) the number of shared genes and interactions between NAD and NSC networks (blue diagrams); the right part represents the intersection between TAD and TSC GCNs.

In Figure 4, we observe the intersection of interactions for healthy and cancer GCNs. For the healthy networks, the number of shared genes is high, but they only share 0.48% of their edges. On the other hand, the TAD and TSC networks share 35% of their interactions. The intersection between cancer GCNs is then 73-fold larger.

The organizational principles that determine the structure in cancer GCNs are more similar than control networks. The observed co-expression program may indicate that the cancer cell suffers a process of de-differentiation, since cancer networks become more alike than the different lung cell types of origin.

The idea that TAD and TSC networks are suffering a de-differentiation process, and can be appreciated from the increase of intersected edges between cancer networks with respect to the normal counterpart, is based on the following premises:

- The gene co-expression program, in particular, the set of higher co-expression interactions, represents a reliable and significant example of the cellular state of a given phenotype.
- The gene co-expression program can be represented by a network, where nodes correspond to genes, and the edges connecting nodes represent a kind of interaction between any couple of genes.
- A gene co-expression interaction can be defined by a certain type of correlation observed between any two genes. In this case, the measurement used to define an interaction is MI.
- The similarity between two networks can be used, to a certain extent, as a proxy to assess the similarity between two gene co-expression phenotypes.
- The TSC cancer network came from the same cells that give form to the normal tissue-derived NSC network. Analogously, the TAD network comes from normal tissue-derived NAD network.
- NSC and NAD networks came from different cell types.

The similarity between tumor GCNs may be explained (at least partially) by a process of cellular de-differentiation. The NSC and NAD networks share little connectivity, but half of the genes are shared. This implies that although they express at least half of the same genes, they do not co-express in the same way; this is probably because they are well-differentiated cells with specialized tasks.

On the other hand, TSC and TAD networks share 76% of genes and 35% of the co-expression pattern. Tumor cells have a lower degree of differentiation and a higher proliferative power. Two tumors of different origin may be more similar to each other than two samples of specialized normal tissue.

### *trans-* Clusters May Play a Crucial Role in the Carcinogenic Process

#### Components With *cis-* Co-Expression Belong to Neighboring Karyobands

Most of the components that form tumor GCNs contain genes from the same chromosome. The genes from each component, in addition to being from the same chromosome, are located in neighboring regions of the chromosomes. Co-expressed genes are usually within the same karyotype band in all chromosomes (*p*-val< 10^−8^ for TAD, and *p*-val<10^−6^ for TSC, Supplementary Material 1B). In other words, the co-expression of neighboring genes is stronger than between distant genes, even within the same chromosome. These *cis-* components are not, however, significantly associated with biological processes in enrichment analysis.

A plausible explanation regarding the mechanisms for which we observed such a decrease in long-range gene couples, and a concomitant elevation of close gene co-expression interactions, could be chromosomal aberrations or the aforementioned copy number alterations (CNAs). This latter could be partially answered by an analysis of copy number alteration data and contrasted that with our network data. Preliminary results in breast cancer have shown that copy number alteration events are not highly correlated with clusters of physically close genes with high co-expression interactions. The complete analysis of CNAs implication in the lung cancer co-expression program is under development.

#### Shared *trans-* Components Are Significantly Associated With Biological Functions

**Figure 5:**
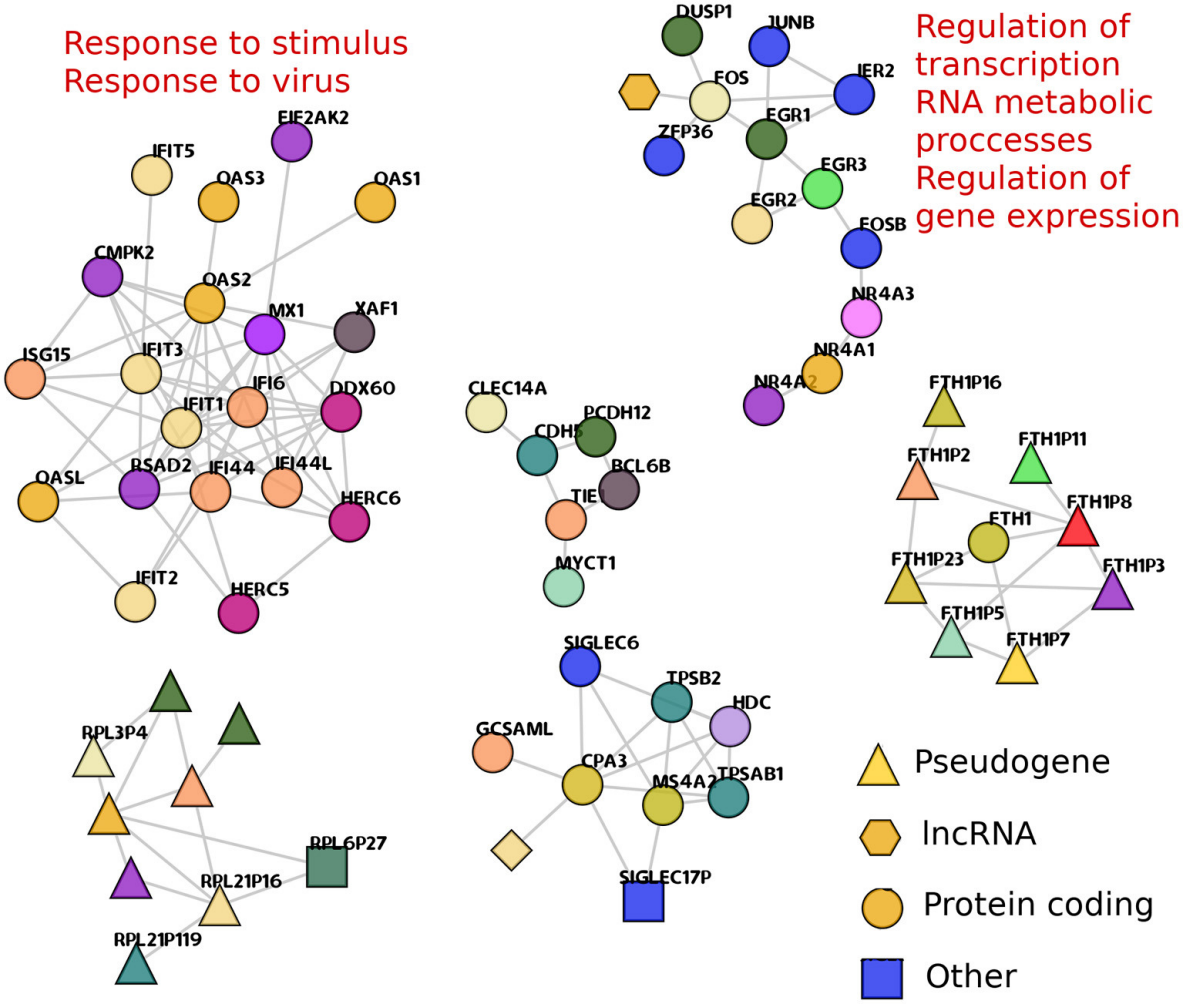
Shared *trans-* components between TAD and TSC networks. *trans-* clusters that are found in both TAD and TSC are depicted. The shape of nodes represents the transcriptional species of the nodes. Enriched processes that are associated with a particular component are presented. The FTH1 component (bottom right) shows one only gene, that codifies for ferritin1, and all other nodes in the component are ferritin pseudogenes, showing that in this case, co-expression favor sequence similarities over gene physical closeness.

Despite the fact that the large majority of gene co-expression interactions in cancer GCNs are *cis-*, a small subset of *trans-* edges appears in both cancer networks. In fact, some *trans-* clusters are also shared between cancer phenotypes. Figure 5 shows the shared *trans-* clusters between the two lung cancer GCNs. Additionally, two of those components are significantly associated with biological processes.

One of them, composed of OAS1, or IFIT genes, resulted enriched in 26 terms (Supplementary Material 2). They are related to processes such as response to virus and response to stimulus. The second enriched *trans-* component (with EGR, FGR, FOSB, and JUNB genes) is associated with the regulation of gene expression, regulation of transcription, and metabolic processes. Thirty-four GO categories resulted enriched for this geneset (Supplementary Material 3).

We previously reported (Alcalá-Corona et al., 2018) a gene co-expression network for HER2+ subtype breast cancer, which contained a component, very similar to the one with IFIT and OAS genes. This component was also associated with viral response. In Alcalá-Corona et al. (2018), additional to the association with virus-related processes, these genes were mostly overexpressed. Here, these genes in TSC network are mostly underexpressed. Moreover, in TAD network, this gene subset is not biased to a particular differential expression trend. The differential expression of all genes in this analysis can be found in Supplementary Material 4.

It is worth to notice that the HER2+ breast cancer network considered there was constructed based on microarray data, and this one is an RNA-Seq-based analysis. Despite technologies are different and also the primary organ in which this gene subset was found, the co-expression associations are the same in a very small group of genes. It is more remarkable that both cases present opposite differential expression trend. This could be another instance in which co-expression features are more robust that gene expression itself.

### Cancer Networks Edges Are Biased to Genes With the Same Differential Expression Trend

Within the Top-10,000 GCN, we observed 5,783 genes for TAD and 5,122 for TSC. Hence, the GCNs do not contain the sufficient number of genes to analyze their whole genome differential expression patterns. To overcome this, we decided to analyze larger GCNs. For that purpose, we conserved gene interactions with a *p* − *value* < 10^−8^ for both cancerous phenotypes.

In the case of TAD, the resulted GCN contains 170,190 interactions and 14,073 genes, which means that almost all genes in the genome participate in the structure of that network. By setting the *Log*_2_*FC* threshold in ±1.5, the number of significant DEGs was 1,056 for overexpressed and 1,304 showed underexpression.

**Figure 6:**
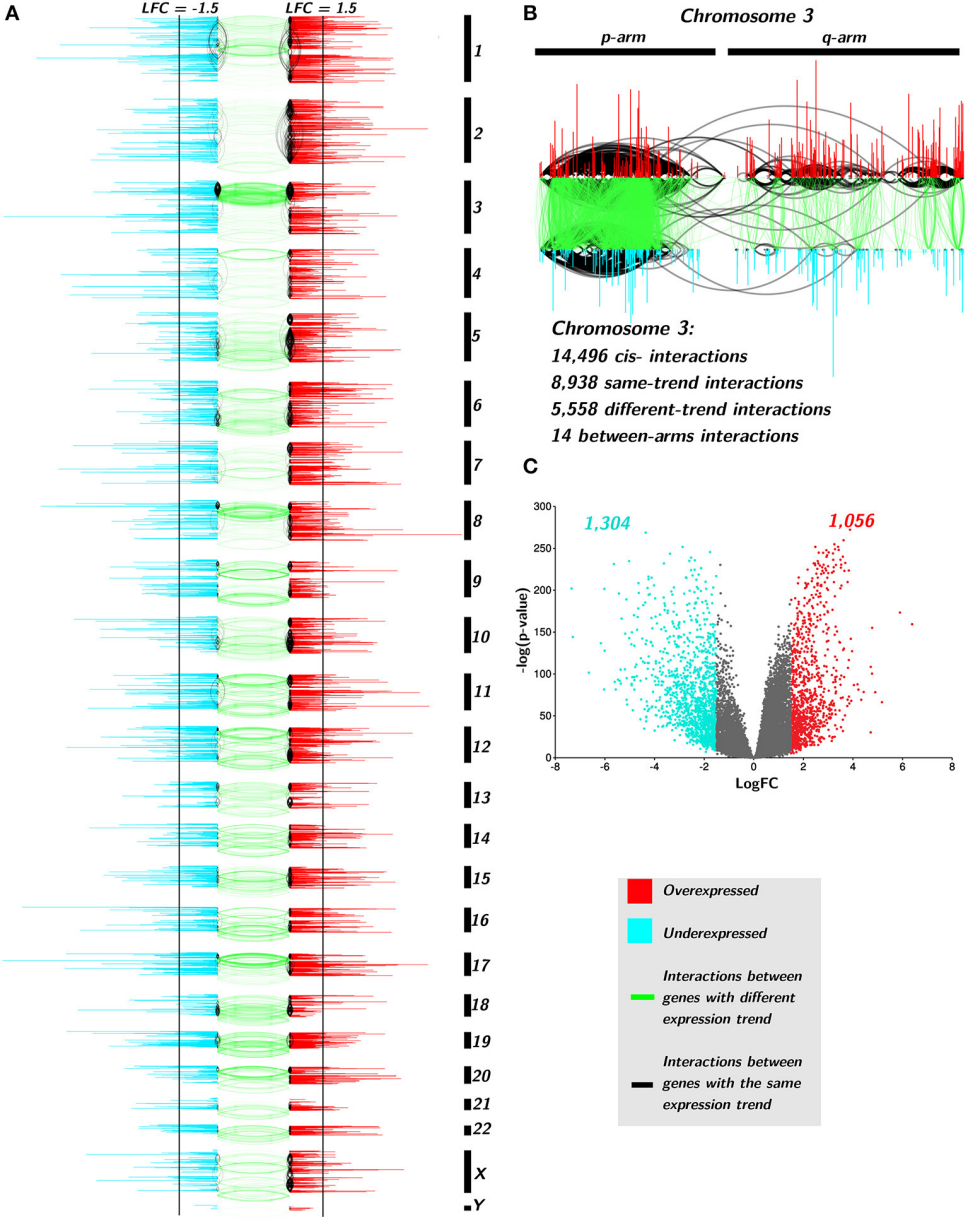
Differential expression trend influences the interactions of TAD network. **(A)** Genes are placed according to its starting base per chromosome. Color of genes takes account for the differential expression trend: red for positive differential expression and turquoise for negative ones. Black vertical lines indicate the threshold point for differential expression (±1.5). Black edges join genes with the same differential expression trend, meanwhile green links represent interactions between different trend genes. **(B)** Zoom-in to Chr3. **(C)** volcano plot for TAD genes with the aforementioned threshold.

In Figures 6A,B, only *cis-* interactions are depicted. Green links join co-expressed genes with an opposite differential expression trend, i.e., one gene presents positive *Log*_2_*FC* and the other one has a negative *Log*_2_*FC*. Black interactions join *cis-* genes that have the same differential expression trend: both genes are over- or underexpressed. There are more underexpressed genes than overexpressed ones (1,304 vs. 1,056, Figure 6C). Additionally, underexpressed genes are more broadly differentially expressed than the overexpressed ones (Supplementary Material 4).

Regarding interactions, there are more black edges (joining same-expression-trend genes) than green ones in all chromosomes (119,574 vs. 40,445, p-val< 10^−8^, Supplementary Material 5). Moreover, the large majority of same-trend interactions occurs between genes with positive*Log*_2_*FC* (110,714) than those with negative differential expression (8,860) in all chromosomes (*p* − *value* < 10^−10^, Supplementary Material 5).

In the case of interactions between negative *Log*_2_*FC* genes, chromosomes 3, 8, and 18, have the majority of intra-trend links. The p-arm of chromosome 3 has dense interactions hotspots in both intra- and inter-trend genes. There is a common deletion in Chr3p in lung cancer (Lerman et al., 2000; Kou et al., 2020). It is known that several tumor suppressor genes are located at 3p (Varella-Garcia, 2010). Partial deletion of 3p occurs in almost all lung carcinomas (Kou et al., 2020). This deletion includes tumor suppressor genes, such as RASSF1 (3p21.3) or TUSC2 (FUS1, 3p21.3) (Kok et al., 1997). These genes are found in the TAD network and both are downregulated.

Another zone with a high number of intra-trend edges is the q-arm of chromosome 10. A deletion in Chr10q24-26 in small cell lung carcinoma has been reported (Petersen et al., 1997; Kim et al., 1998). PTEN gene is located on 10q23.3 and it is also present in the network, downregulated but non-significantly underexpressed. Alterations in PTEN have been reported in around 20% of SCLC (Yokomizo et al., 1998). Despite this analysis was performed on nonsmall cell lung carcinomas, the intra-trend interactions hotspot observed in Chr10 could be associated with chromosomal-level events in NSCLC.

*cis-* interactions between genes that belong to different arms are also scarce. In the top right part of Figure 6, the zoom in of Chr3 shows that from almost 15,000 *cis-* Chr3 gene co-expression relationships, only 14 appear between genes from different arms, and none of them are between different expression trend genes.

All of these results appear to indicate that in NSCLC, the co-expression landscape is dominated by physically close genes. These genes, in turn, share other characteristics, such as the differential expression pattern. At this point, we do not know the functional causes behind this phenomenon.

In the case of TSC, the difference between same-arm co-expression interactions as compared to those in different-arm ones is even larger. The total number of significant interactions for TSC is 232,355. Intra-arm *cis-* interactions are 222,839, i.e., the 95.9% of all interactions. The *trans-* interactions cover 9,081, the 3.9% of all interactions. The inter-arm *cis-* interactions are only 435. The fact that for TSC network, we observed 20 times fewer interactions between inter-arm *cis-* relationships than *trans-* interactions, which was unexpected. The latter may suggest that some *trans-* interactions are crucial to maintain certain processes in the tumor cell. In Supplementary Material 6, we provide the Cytoscape session .cys file containing all networks used in this work.

### Loss of *trans-* Co-Expression in Cancer Does Not Depend on the Network Size or the Number of Samples

As mentioned in section 2, we analyzed the GCNs with the top 10,000 interactions. To assess the validity of the results shown here, we decided to carry out calculations for a broader range of interactions, from the top 1,000 edges to the top 100,000, i.e., three orders of magnitude, to evaluate whether or not, the differences in the *cis-/trans-* proportion were maintained.

Supplementary Material 7 shows the proportion of *cis-* interactions of the total of edges at different cutoff values. As it is noted, the proportion of this imbalance in lung cancer networks is essentially preserved independent of the cutoff value. This confirms our assertion that the fundamental phenomenon we are observing, regarding structural features of GCNs, is indeed maintained over a fairly wide range of interaction cutoffs.

We commented above that the number of cancer samples is much larger than healthy ones (~ 1,000 vs. 100). To assure that the obtained results for cancer networks were not due to the sample size, we generated 10 randomized expression matrices with 100 samples for adenocarcinoma tumors and other 10 matrices for squamous cancer data. The GCNs obtained with this method were pruned to 10,000 interactions and calculated its *cis/trans* proportion.

Supplementary Material 8 contains a Cytoscape session file, including the 20 different realizations of randomized networks, 10 for TAD and 10 for TSC. There it can be observed that with 100 random samples, the effect of loss of *trans-* co-expression prevails in all instances.

## Conclusions

As a summary of findings, in this work we have shown that:

- gene co-expression networks in lung cancer suffer a dramatic loss of distant interactions;
- adenocarcinoma and squamous cell lung cancer GCNs are much more alike (in terms of gene interactions) than the networks formed by adjacent-to-tumor normal-derived tissue;
- the co-expression interactions in lung cancer are biased to appear in genes that are in the same chromosome;
- in lung cancer, interactions occur preferably between genes from the same cytoband;
- top gene interactions in lung cancer occur often between genes with the same differential expression trends, in special between upregulated genes;
- shared *trans-* (inter-chromosome) connected components are strongly associated with important biological functions such as immune response and regulation of gene transcription;
- these features has been observed for the first time in lung tissue-derived GCNs.

We have observed an important intersection between genes and links in lung cancer networks, which is opposed to the observed in healthy lung tissue-derived networks. This finding leads us to suggest that a de-differentiation mechanism appears during lung carcinogenesis.

The networks used in this work were inferred from lung cancer samples with no other filter than the type of lung cancer (adenocarcinoma and squamous cells). Further investigation in this line of research must be focused on constructing and infer networks based on progression stages of these types of cancer to observe whether or not later stages are more similar than the early ones.

We strongly believe that the current knowledge regarding gene co-expression and the concomitant functional regulation of the transcriptional program in cancer phenotypes will be improved and better understood by aggregating other omic layers to these systems. Furthermore, the effect of loosing the long-range co-expression observed in more than one cancer tissue (breast, kidney, and now lung) may be an instance of a more complicated phenomenon that could be behind of a novel—not yet described—hallmark of cancer.

In any case, the present results contribute to advancing our knowledge of the deep intricacies behind transcriptional regulation in cancer. This, in turn, will be helpful not only to establish better the basic foundations of cancer biology but also to devise ways in which this knowledge may be translated into diagnostics, prognostics, and therapies for lung cancer patients.

## Data Availability Statement

The original contributions presented in the study are included in the article/Supplementary Material, further inquiries can be directed to the corresponding author/s.

## Author Contributions

SA-E performed calculations, analyzed the data, and wrote the paper. JZ-F performed calculations and wrote the code. JE-E co-conceived the project, analyzed the data, made the figures, and wrote the paper. EH-L co-conceived the project, coordinated the theoretical sections, analyzed the data, and wrote the paper. All authors read and approved the final manuscript.

## Conflict of Interest

The authors declare that the research was conducted in the absence of any commercial or financial relationships that could be construed as a potential conflict of interest.

## Supplementary Material

Supplementary Material 1A Number of *cis-* interactions for the four networks.

Supplementary Material 1B Number of interactions within genes from the same karyotype band (intra-karyoband) and different karyoband (inter-karyoband) per chromosome.

Supplementary Material 2 Over-representation analysis of shared *trans-* cluster composed of OASL1 and IFIT, among other genes. The output of this file correspond to the standard output of g:ProfileR web interface.

Supplementary Material 3 Over-representation analysis of shared *trans-* cluster composed of FOSB and JUN, among other genes.

Supplementary Material 4 Differential expression values for TAD and TSC samples.

Supplementary Material 5 Table with the number of same trend positive interactions, same trend negative interactions, and different trend interactions in all chromosomes.

Supplementary Material 6 Cytoscape .cys file containing all networks inferred for this study.

Supplementary Material 7 Loss of *trans-* co-expression is not dependent of the network size. In this plot, we provide the proof that network size does not influence the effect of loss of inter-chromosome interactions proportion in lung carcinoma. Different network cutoffs were calculated for this purpose. Note that 1,000 to 100,000 top interactions given on X-axis. On Y-axis, the *cis-* proportion is represented, i.e., the number of *cis-* interactions over the total interactions in said cutoff. Green and yellow dots represent tumor networks, whereas blue and orange ones take account for normal networks.

Supplementary Material 8 Cys file with cancer networks obtained by random selection of 100 samples.
