# Supplementary figures and images for: Loss of Long Distance Co-Expression in Lung Cancer
Source: Front Genet. 2021 Mar 10;12:625741. doi: 10.3389/fgene.2021.625741 (PMC7987938; doi:10.3389/fgene.2021.625741)

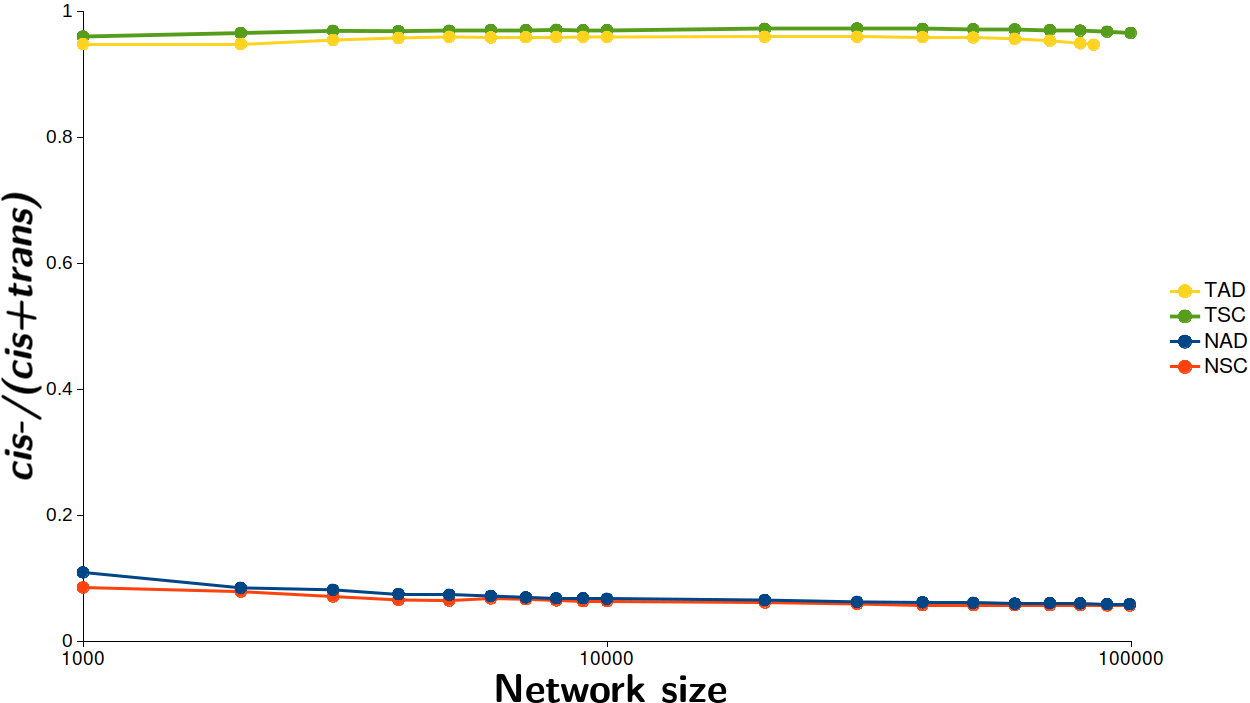

Supplement: Supplementary Material 7 — Loss of trans- co-expression is not dependent of the network size. In this plot, we provide the proof that network size does not influence the effect of loss of inter-chromosome interactions proportion in lung carcinoma. Different network cutoffs were calculated for this purpose. Note that 1,000 to 100,000 top interactions given on X-axis. On Y-axis, the cis- proportion is represented, i.e., the number of cis- interactions over the total interactions in said cutoff. Green and yellow dots represent tumor networks, whereas blue and orange ones take account for normal networks. [file Image_1.PNG]
